# Kaolinite Nanocomposite Platelets Synthesized by Intercalation and Imidization of Poly(styrene-*co*-maleic anhydride)

**DOI:** 10.3390/ma8074363

**Published:** 2015-07-16

**Authors:** Pieter Samyn, Gustaaf Schoukens, Dirk Stanssens

**Affiliations:** 1 Chair for Bio-based Materials Engineering, Faculty for Environment and Natural Resources, Freiburg Institute for Advanced Studies (FRIAS), University of Freiburg, Werthmannstrasse 6, D-79085 Freiburg, Germany; 2 Department of Textiles, Ghent University, Technologiepark 907, B-9052 Zwijnaarde, Belgium; E-Mail: Gustaaf.Schoukens@UGent.be; 3 Topchim N.V., Nijverheidstraat 98, B-2160 Wommelgem, Belgium; E-Mail: info@topchim.be

**Keywords:** kaolinite, guest displacement, poly(styrene-*co*-maleimide), nanoparticles

## Abstract

A synthesis route is presented for the subsequent intercalation, exfoliation and surface modification of kaolinite (Kln) by an imidization reaction of high-molecular weight poly(styrene-*co*-maleic anhydride) or SMA in the presence of ammonium hydroxide. In a first step, the intercalation of ammonolyzed SMA by guest displacement of intercalated dimethylsulfoxide has been proven. In a second step, the imidization of ammonolyzed SMA at 160 °C results in exfoliation of the kaolinite layers and deposition of poly(styrene-co-maleimide) or SMI nanoparticles onto the kaolinite surfaces. Compared with a physical mixture of Kln/SMI, the chemically reacted Kln/SMI provides more efficient exfoliation and hydrogen bonding between the nanoparticles and the kaolinite. The kaolinite nanocomposite particles are synthesized in aqueous dispersion with solid content of 65 wt %. The intercalation and exfoliation are optimized for a concentration ratio of Kln/SMI = 70:30, resulting in maximum intercalation and interlayer distance in combination with highest imide content. After thermal curing at 135 °C, the imidization proceeds towards a maximum conversion of the intermediate amic acid moieties. The changes in O–H stretching and kaolinite lattice vibrations have been illustrated by infrared and FT-Raman spectroscopy, which allow for a good quantification of concentration and imidization effects.

## 1. Introduction

Approximately 40% of the produced kaolinite (Kln) is used as filler agents in the bulk or surface coating of paper. In filling, the kaolinite is mixed with the pulp and forms an integral part of the paper sheet to give it body, colour and opacity [1]. In coating, the kaolinite is applied on the paper surface along with a binder to improve gloss, colour, high opacity, and printability [2]. The plate-like structure of kaolinite makes it particularly interesting to be used as an additive that improves the barrier properties [3], as the layered structure causes a tortuous path retarding the diffusion of air and gasses. In parallel, hydrophobization of the individual kaolinite layers might stabilize them in aqueous dispersion and further improve the resistance against water penetration through the coating. The surface decoration of kaolinite platelets with hydrophobic nanoparticles increases the surface hydrophobicity by creating a micro- to nanoscale hierarchical structure [4]. In view of these applications, the intercalation and exfoliation of kaolinite and subsequent precipitation of polymeric nanoparticles may provide interesting nanocomposite additives, if they can be delivered in a stable aqueous environment with high solid content. In contrast, most studies of hybrid clay composites resulted in limited loadings of about 10 wt % [5]. The strong interactions between the individual clay mineral layers mainly impede successful exfoliation at high concentrations, while the dispersion depends on the nature and amount of surfactants [6]. The intercalation [7] and surface coating of clay minerals with nanoparticles [8,9] may help to increase the solid content for paper coating applications. 

The intercalation of kaolinite is mostly hindered by strong hydrogen bonding between the individual layers of the clay mineral, and is less evident than for montmorillonite [10], or swelling smectite [11]. The strong cohesion between adjacent kaolinite layers results from its asymmetric structure with siloxane at one side and aluminol groups at the other side creating permanent dipole interactions. Only certain molecules and ions of short chain organic acids with suitable size can penetrate within the interlayer and break the hydrogen bonds. A limited number of dipolar organic solvents such as dimethylsulfoxide (DMSO) [12], hydrazine [13], formamide [14], N-methylformamide [15], dimethyl-ormamide [16], acetamide [17], urea [18], or potassium acetate [19] can be intercalated directly and as co-intercalants [20]. The complete intercalation of kaolinite is mostly obtained in combination with mechanochemical processes [21]. The different reactive molecules can be classified according to the interaction mechanism by hydrogen bonding, dipole formation or ionic interactions [22]. More recently, the interlayer expansion was successful in the presence of ionic liquids [23], or vegetable oils [24]. Otherwise, various organic species can be inserted by so-called “guest displacement” of the intermediate intercalate and insertion of new guest molecules. As such, Kln/methanol was synthesized by guest displacement reaction of a Kln/N-methylformamide, and this intermediate intercalation compound was further used for insertion of alkylamines and water [25]. Traditionally, the Kln/DMSO intermediates are used for the intercalation of, e.g., methyl-pyrrolidone [26]. The stability and deintercalation properties of intermediate Kln/DMSO were followed by a decrease in intensity of spectral absorption bands related to hydroxyl and CH stretching [27]. As such, cyclic imides could be inserted from an aqueous solution by complete interchange of the DMSO in relatively short times [28]. The organically pre-modified kaolinite can also be used for the intercalation of larger molecules such as poly(ethylene glycol) [29], poly(ethylene oxide) [30], polyhydroxybutyurate [31] or nylon-6 [32]. For synthesis of hybrid organic-inorganic clay nanocomposites, the intercalation and adsorption of polymeric monomers can be followed by an *in-situ* free radical solution polymerization, as done for polystyrene and maleic anhydride [33]. After formulation of organically modified montmorillonite, a good dispersion of amide acid and imide could be observed while the presence of aliphatic quarternary ammonium affected imidization and solvent removal during thermal treatment [34]. 

The full exfoliation of kaolinite into individual silicate layers can been achieved by additional stimuli such as microwave irradiation [35], and is further enhanced under external mechanical forces by ball-milling, or high shear mixing [36]. The simultaneous acidic treatment supports the ability for mechanical exfoliation, contributing to a narrow particle size distribution and high surface areas [37]. The exfoliation by thermally controlled low-temperature washing procedures is more efficient than milling and yields kaolinite with interstratified layers of grafted urea and water [38]. A one-step route for exfoliation of kaolinite has been developed by intercalation of ammonium salts and solvent swelling [39]. 

**Figure 1:**
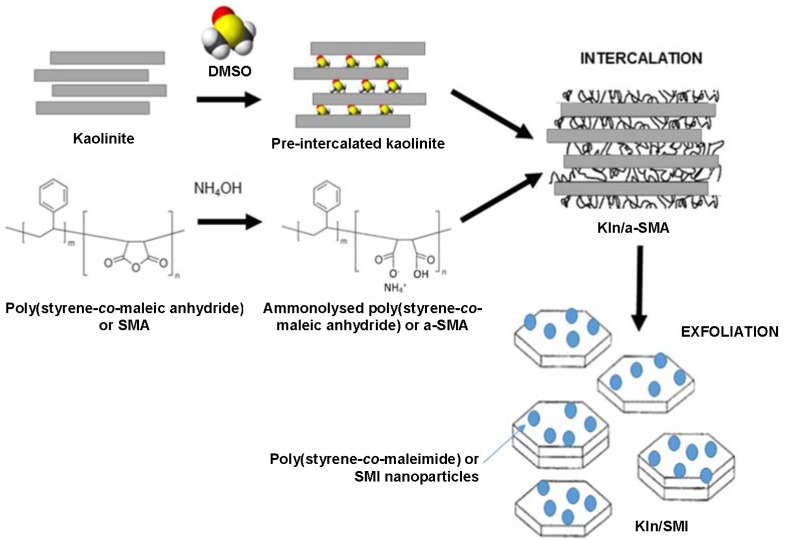
Concept for intercalation of kaolinite with poly(styrene-*co*-maleic anhydride) and exfoliation by imidization into poly(styrene-*co*-maleimide) or SMI nanoparticles.

In this work, a one-step method for intercalation, exfoliation and surface modification of kaolinite is presented, leading to stable aqueous dispersions with high solid content of hybrid organic-inorganic kaolinite nanocomposite platelets. Schematically (Figure 1), a high-molecular weight copolymer of ammonolyzed poly(styrene-*co*-maleic anhydride) or a-SMA is intercalated by guest displacement of DMSO and poly(styrene-*co*-maleimide) or SMI n anoparticles are subsequently formed by imidization. A similar approach has previously been introduced for the synthesis of organic-inorganic pigments by direct reaction of a kaolinite slurry (polyacrylic dispersant) with SMA [40], but intercalation or exfoliation had not occurred. In the present work, the latter features can be obtained in addition to the deposition of SMI nanoparticles by implementing appropriate pre-intercalation conditions. In future applications, the exfoliated kaolinite particles with hydrophobic nanoparticles deposits on the surfaces can be incorporated as pigments for protective barrier coatings on papers. As the main aim of this paper lies in the optimization of the synthesis route and full characterization of the pigments, the coating application and characterization will be covered in a following study.

## 2. Results and Discussion

### 2.1. Intercalation and Exfoliation of Kaolinite

**Figure 2:**
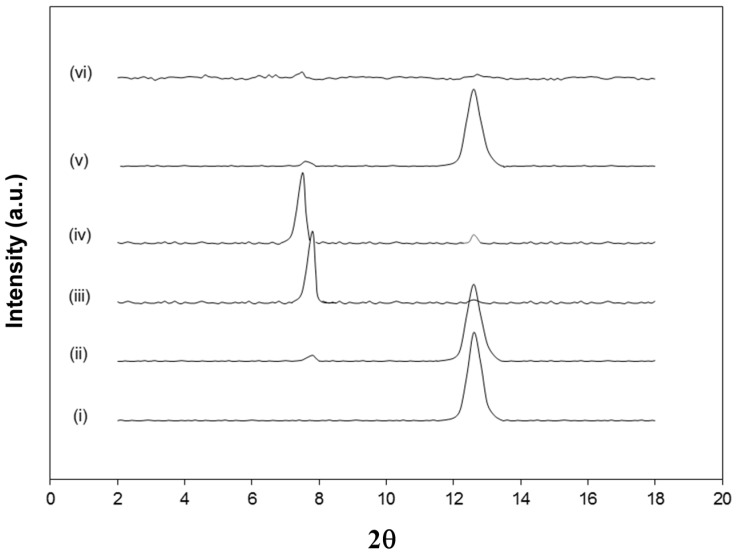
X-ray diffraction (XRD) powder diffraction patterns of (**i**) Kln, (**ii**) Kln/DMSO 20 h, (**iii**) Kln/DMSO + water 10 h, (**iv**) Kln/a-SMA = 70:30, (**v**) Kln/SMI = 70:30 physically mixed (**vi**) Kln/SMI = 70:30 chemically reacted.

The intermediate intercalated kaolinite with either DMSO (Kln/DMSO) or DMSO + water (Kln/DMSO + water) was prepared and subsequently used as a host material for the intercalation of ammonolysed SMA (Kln/a-SMA), which is later transformed by imidization into hybrid nanocomposite kaolinite platelets with deposited SMI nanoparticles (Kln/SMI). The powder X-ray diffraction (XRD) patterns of Kln, Kln/DMSO, Kln/DMSO + water, Kln/a-SMA and Kln/SMI in a ratio 70:30 (Figure 2), indicate different arrangements of the kaolinite layers after diffusion of the polymer molecules in the interlayers. The original kaolinite has a single sharp diffraction peak at 2θ = 12.4° corresponding to the (001) basal planes with a spacing of *d*_001_ = 0.725 nm. The intercalation with pure DMSO seems inefficient after 20 h sonication, as the main diffraction peak for Kln/DMSO is comparable to original Kln and only a very small diffraction peak at a lower diffraction angle of 2θ = 7.9° is observed. The intercalation with DMSO + water after 10 h sonication is much more efficient as observed by the appearance of a clear diffraction peak at 2θ = 7.9° corresponding to *d*_001_ = 1.12 nm, which represents a lattice expansion *d* = 0.40 nm relatively to the original Kln. After replacing the DMSO + water with a-SMA, the diffraction peak shifts to a minimum of 2θ = 7.7° as an indication that the kaolinite lattice structure was further expanded to *d*_001_ = 1.23 nm in presence of ammonolyzed copolymer (KAO/a-SMA = 70:30). This data illustrates that high-molecular weight SMA has penetrated in between the kaolinite layers and displaces the initial DMSO, which leads to further expansion of the kaolinite layers over a distance of *d* = 0.51 nm relative to the original kaolinite. The absence of DMSO after precipitation and several washing steps will be further evidenced by spectroscopy. The displacement of DMSO as guest molecules by another intercalating agent is generally known as a fast and efficient method compared with the direct intercalation of molecules [28], but the successful displacement of DMSO by high-molecular weight SMA molecules is not evident compared with the intercalation of regular low-molecular weight species. For Kln/a-SMA, a very small residual diffraction peak at 2θ = 12.4° might correspond with pure kaolinite and relate to a portion of non-intercalated species (e.g., it appears also very slightly in Kln/DMSO + water) or partial deintercalation during washing.

The intercalation ratio (I.R.), interlayer distance (*d*_001_) and interlayer distance expansion (*d*) are calculated for different ratios KAO/a-SMA from the XRD patterns. The intercalation ratio (I.R.) is defined in Equation (1):

(1) $$\text{I}.\text{R}.\;\left(\%\right)=\;\frac{I_{i\left(001\right)}}{I_{i\left(001\right)}+\;I_{k\left(001\right)}}\;\times \;100$$

with *I_i_*_(001)_ the peak intensity corresponding to the intercalated kaolinite phase and *I_k_*_(001)_ the peak intensity for the residual kaolinite phase [31]. The data for *d*_001_ spacing of Kln/a-SMA prepared from the Kln/DMSO + water pre-intercalates (Table 1) show that the best intercalation is obtained for a ratio Kln/a-SMA = 70:30, where the intercalation ratio is at around 90% and the layers have expanded to a maximum distance of 1.234 nm. Therefore, this particular concentration will be further used and studied in most detail. For the other concentration ratios, the expansion gradually increases and the intercalation ratio is relatively high for Kln/a-SMA = 95:5 to 70:30, while the expansion remains smaller and comparable to Kln/DMSO + water for the higher concentrations of Kln/a-SMA. For the applied intercalation duration of 10 h, the intercalation of Kln/DMSO + water was only partial but it further developed during treatment with a-SMA. There is an experimental optimum concentration for the intercalant, as the lower contents of a-SMA have not yet fully occupied the interlayer space and the higher concentrations do not efficiently intercalate as they rather form a polymer layer (see microscopy) and the Kln/DMSO + water intercalation remains predominant. A rationale for the optimum intercalant concentration can be found, as (i) it might be difficult for the macromolecules to diffuse in between the non-intercalated platelets, while (ii) the diffusion is hindered when the intercalation density becomes too high. In addition, the interactions with high-molecular weight SMA might be influenced by the specific molecular configuration and tendency for self-assembly [41].

The XRD pattern of Kln/SMI 70:30 after chemical imidization shows no diffraction peaks related to the *d*_001_ spacing. Based on these data, the formation of SMI has induced a re-arrangement of the kaolinite layers along the (001) direction: the characteristic *d*_001_ spacing disappears because the crystallographic order is lost with complete exfoliation [42]. It is known that the exfoliation of kaolinite mainly affects the *c*-axis (*d*_100_) and not the *a*- and *b*-axis [39]. One reason for the exfoliation of Kln/SMI during the chemical imidization is attributed to the highly viscous state that is created during reaction, which results in high shear forces in the reaction mixture over the first 3 h of reaction. Similar reaction profiles have been previously described for the synthesis of pure SMI [43], with an initial increase in viscosity due to the presence of ammonolysed a-SMA, followed by a drop in viscosity upon imidization. The high internal shear forces seems to efficiently contribute to the intercalation and exfoliation of the kaolinite. The absence of diffraction peaks in XRD patterns was mainly observed for Kln/SMI = 70:30, while the other ratios Kln/SMI = 95:5, 90:10, 80:20 and 50:50 did show some more residual peaks of unreacted kaolinite. In contrast, the physical mixing of kaolinite and SMI is not efficient for intercalation or exfoliation and the original kaolinite structure is not modified. The evidence for exfoliation of Kln/SMI from XRD spectra is better than obtained in previous studies starting from kaolinite slurry [40], where the peaks related to the basal planes were still present in the Kln/SMI compounds thus with the same *d*_001_ spacing as present in the native kaolinite.

**Table 1:** Summary of the intercalation properties for Kln/a-SMA including intercalation ratio (I.R.), interlayer distance *d*_100_ and interlayer distance variation (*d*).

Material	I.R. (%)	*d*_001_ (nm)	*d* (nm)
Kln	-	0.725	-
Kln/DMSO	5.2	0.725	0
Kln/DMSO + water	84.3	1.123	0.398
Kln/a-SMA = 95:5	89.4	1.130	0.405
Kln/a-SMA = 90:10	89.2	1.152	0.427
Kln/a-SMA = 80:20	90.2	1.185	0.460
Kln/a-SMA = 70:30	93.2	1.234	0.509
Kln/a-SMA = 50:50	74.2	1.128	0.403
Kln/a-SMA = 30:70	64.2	1.125	0.400
Kln/a-SMA = 20:80	60.2	1.126	0.401
Kln/a-SMA = 10:90	60.1	1.128	0.403
Kln/a-SMA = 5:95	55.4	1.128	0.403

### 2.2. Intercalation Kaolinite with Ammonolyzed a-SMA

**Figure 3:**
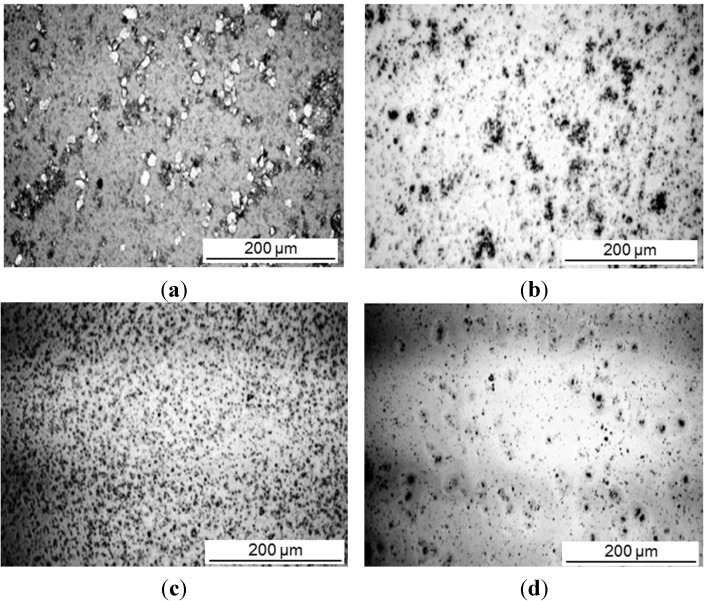
Optical microscopy representing size distribution of intercalated kaolinite particles, (**a**) Kln/DMSO + water, (**b**) Kln/a-SMA = 80:20, (**c**) Kln/a-SMA = 70:30, (**d**) Kln/a-SMA = 50:50.

**Figure 4:**
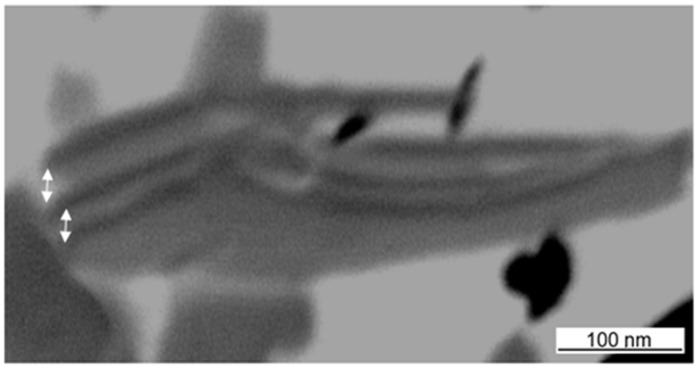
TEM analysis of an intercalated kaolinite platelet with Kln/a-SMA = 70:30, showing the kaolinite layers and increment in interlayer distance (arrows).

The intercalation was investigated for different concentrations of a-SMA and pre-intercalated Kln/DMSO + water. The size reduction of the original kaolinite powder after sonication was a preliminary indication for the efficient interaction. Therefore, optical microscopy pictures of different mixing ratios Kln/a-SMA were taken after sonication (Figure 3). A homogeneous dispersion without agglomeration is obtained at ratio Kln/a-SMA = 70:30 resulting in the formation of fine particles. The lower amounts of a-SMA resulted in coarse (unreacted) kaolinite particles while the higher amounts of a-SMA resulted in film formation of the excess SMA wherein the kaolinite particles are inhomogeneously dispersed. A sample with Kln/a-SMA = 70:30 after intercalation was evaluated by transmission electron microscopy (TEM) analysis (Figure 4), confirming expansion of the individual kaolinite layers that appear as dark phase contrast. In literature, it is well known that because of the structural differences between the basal surfaces of individual kaolinite layer, after exfoliation, there is a tendency for the formation of scrolls [39]. This behavior is not observed here, as the scrolling highly depends on the used (pre-)intercalant and the a-SMA used in present case is known to act as a good dispersant in aqueous dispersions due to charge effects, which obviously also reduces the interactions between the individual kaolinite layers. 

**Figure 5:**
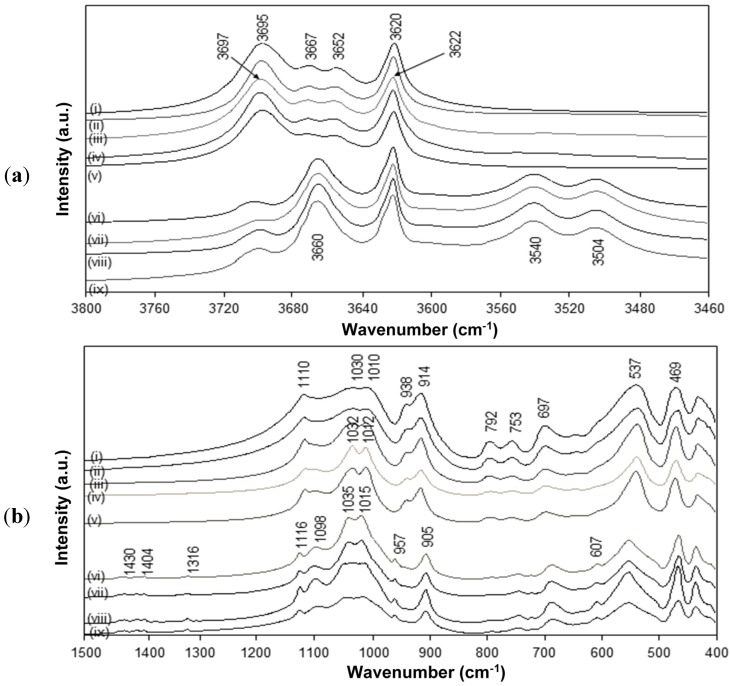
FTIR spectra characteristic for (**a**) O–H stretching region, (**b**) lattice vibrations of (i) Kln, (ii) Kln/DMSO 10 h, (iii) Kln/DMSO 20 h, (iv) Kln/DMSO + water 10 h, (v) directly intercalated Kln/a-SMA* = 70:30, (vi) Kln/a-SMA = 80:20, (vii) Kln/a-SMA = 70:30, (vii) Kln/a-SMA = 50:50, (ix) Kln/a-SMA = 30:70.

The FTIR spectra for intercalated kaolinite have been well characterized before and are characteristic for the interlayer conformation [44]. The spectra for native kaolinite, pre-intercalant Kln/DMSO and some Kln/a-SMA intercalates are further evaluated (Figure 5). Residual absorption bands of DMSO have not been observed after washing the samples, confirming complete removal of the DMSO and displacement by a-SMA. A directly intercalated Kln/a-SMA* with ratio 70:30 is included as reference and indicates unsuccessful intercalation. The spectral variations for Kln/a-SMA indicate significant changes in O–H stretching region (Figure 5a) and kaolinite lattice vibration region (Figure 5b), which provides evidence for good intercalation of a-SMA. The successful intercalation of a-SMA is described in terms of variations in vibrational modes of the kaolinite structure and is fully detailed in Supplementary Materials.

**Figure 6:**
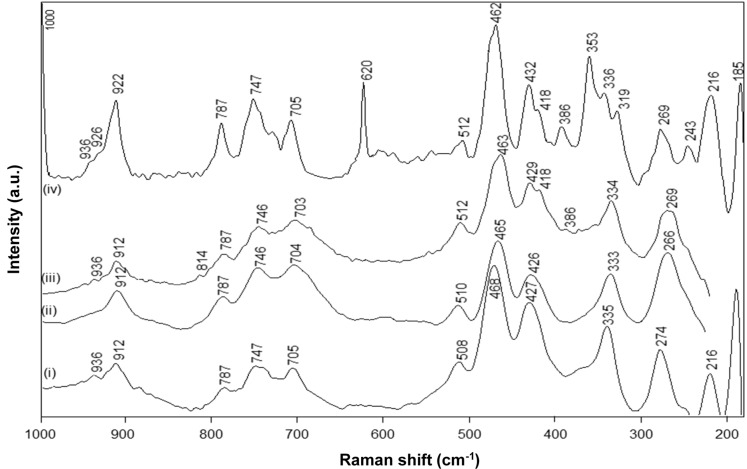
FT-Raman spectra for (**i**) Kln, (**ii**) Kln/DMSO 20 h, (**iii**) Kln/DMSO + water 10 h, (**iv**) Kln/a-SMA = 70:30.

The FT-Raman spectra for native kaolinite, pre-intercalant Kln/DMSO and a Kln/a-SMA intercalate at ratio 70:30 (Figure 6) illustrate the typical fingerprint region of the kaolinite lattice structure between 200 and 1000 cm^−1^ [44,45]. The absence of DMSO bands at 666 and 696 cm^−1^ confirms that the intercalated kaolinite has been efficiently washed. The spectra for native Kln and intercalated Kln/a-SMA 70:30 show significant changes in the low frequency vibrational modes corresponding to the kaolinite structure, while almost no spectral variations for Kln/DMSO and only partial variations for Kln/DMSO + water are observed. In agreement with previous FTIR spectra, the presence of water favours the intercalation but the time of 10 h was still too low to induce complete intercalation. The partial pre-intercalation with DMSO + water was, however, necessary to promote the subsequent intercalation of a-SMA by guest displacement. The following spectral regions can be distinguished for the kaolinite structure:

- The band region at 920–900 cm^−1^ is attributed to the Al–OH liberation modes of pure kaolinite: a first band at 912 cm^−1^ is attributed to the position of outer hydroxyl groups and the weaker second band at 936 cm^−1^ is due to the vibration of inner hydroxyl groups [46]. The first band shows a significant increase in intensity and shifts towards 922 cm^−1^ upon intercalation of Kln/a-SMA, while it remains constant for Kln/DMSO and Kln/DMSO + water. A similar upwards shift in the 912 cm^−1^ band to a new band at 926 cm^−1^ was seen upon intercalation of potassium acetate [46]. This is in contrast with the presence of urea, where a downwards shift in this band region was attributes to the loss of hydrogen bonding within the interlayer [47]. There is typically an additional weak band at 898 cm^−1^ for pure kaolinite that vanished upon intercalation by occupation of the hydroxyl groups. Therefore, the liberation of Al–OH groups in the present situation with an a-SMA intercalant is clearly demonstrated and the upwards shift in wavenumber position indicates the breaking of hydrogen bonds in the kaolinite interlayer and creation of new hydrogen bonds with the intercalant. The second band at 936 cm^−1^ remains existing in the intercalate as the inner hydroxyl groups are not accessible for intercalation, while an additional band at 926 cm^−1^ in Kln/a-SMA corresponds to the additional Al–OH liberation of the outer hydroxyls that are likely not occupied by hydrogen bonding with the intercalant.
- The bands at 787 cm^−1^ (OH-translational region) and 747, 705 cm^−1^ (Si–O–Al translation) for kaolinite are evidently transformed into better resolved bands for the intercalated a-SMA structure, with no significant shift in wavenumber, as also previously observed for potassium acetate [46], or urea intercalant [47]. The region at 400–520 cm^−1^ (Si–O symmetric stretching) has typically two main bands at 427 and 468 cm^−1^ (SiO_4_ tetrahedron) with an additional band at 508 cm^−1^ (SiO_4_ tetrahedron) for kaolinite. This zone becomes more complicated and splits into multiple bands after intercalation, due to the symmetry loss of the Si_2_O_5_ units, as generally seen in silicate glasses [48]. As a result, the band at 462 cm^−1^ increases in intensity while a decrease in intensity and shift for the 512 cm^−1^ band is observed by intercalation, together with the occurrence of additional bands at 429–432 cm^−1^ (SiO_4_ tetrahedron), and 418 cm^−1^: similar changes have been detected for urea and potassium acetate intercalates [47]. The latter can be explained by the equivalence of the outer hydroxyl groups, *i.e.*, the differences in outer hydroxyl groups are removed by formation of the intercalate. The appearance of new bands at 386 and 353 cm^−1^ upon intercalation are also related to the creation of an additional stretching mode in the SiO_4_ tetrahedron and agrees with the additional freedom [49]: these bands confirm that the hydrogen bonds between the Si–O tetrahedra and the outer hydroxyl groups of the next adjacent kaolinite layer have been broken by intercalation. The 386 cm^−1^ band already develops weakly for Kln/DMSO + water, but becomes fully clear for Kln/a-SMA = 70:30. The bands at 243 and 269 cm^−1^ in Kln/a-SMA = 70:30 are attributed to the O–H–O stretching of the triangular kaolinite structure, which might be perturbed through additional interactions and hydrogen bonding with a-SMA, resulting in a new band at 243 cm^−1^ for the intercalate. The low frequency bands at 216 and 185 cm^−1^ related to the AlO_6_ octahedron do not change by intercalation [47]. In general, the complex spectra for intercalated Kln/a-SMA = 70:30 at 300–500 cm^−1^ fundamentally illustrates that single kaolinite layers have been created by intercalation, where the equivalence of the Si–O bonds is no longer present. Finally, the occurrence of new single bands at 620 and 1000 cm^−1^ for Kln/a-SMA corresponds to our previous characterization of the a-SMA copolymers [41], and represents the permanent presence of styrene moieties after washing that confirm the successful intercalation of poly(styrene-*co*-maleic anhydride).

**Figure 7:**
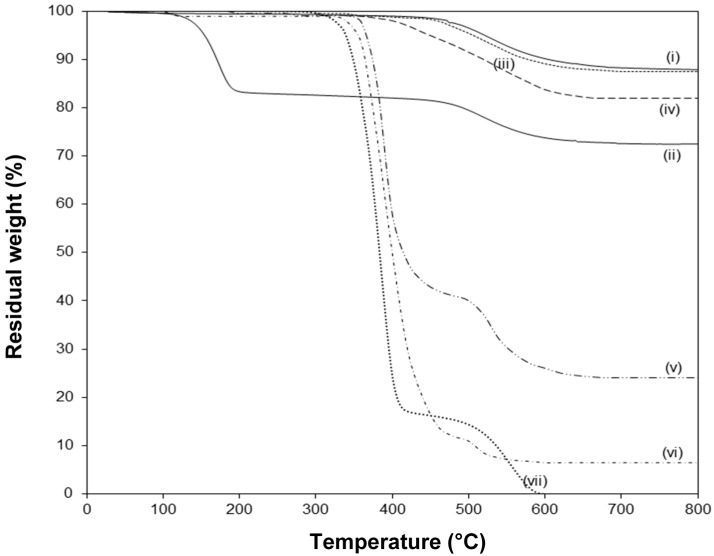
Thermogravimetric analysis (TGA) in air atmosphere of (**i**) pure Kln, (**ii**), Kln/DMSO + water 10h, (**iii**) Kln/a-SMA = 80:20, (**iv**) Kln/a-SMA = 70:30, (**v**) Kln/a-SMA = 50:50, (**vi**) Kln/a-SMA = 30:70, (**vii**) pure SMA.

The thermal stability of native and modified kaolinite is evaluated by TGA (Figure 7). The pure Kln has a single degradation temperature at around 520°C with 12.4% weight loss that can be assigned to the loss of structural water by dehydroxylation of the crystal lattice. The absence of any weight loss at lower temperatures illustrates that the native kaolinite was fully dried. The pre-intercalated Kln/DMSO + water shows a supplementary first degradation step at around 185 °C due to the loss of organic moieties of the intercalate, in parallel with literature data [28]. On the other hand, the main degradation temperature of pure SMA is at 380 °C, and a plateau value at higher temperatures can be attributed to the stability by oxidative crosslinking of the copolymer structure in air [50,51]. The thermal stability for Kln/a-SMA intercalates in different ratios is situated in between the properties of pure Kln and SMA, not showing any trace of DMSO degradation. This proves that the DMSO was successfully removed from the intercalate and displaced by a-SMA: (i) at low ratios Kln/a-SMA = 30:70 and 50:50, the SMA gains thermal stability in presence of kaolinite as a filler material, (ii) at high ratios Kln/a-SMA = 70:30 and 80:20, the intercalated kaolinite has somewhat lower thermal stability than pure Kln but is much better than the Kln/DMSO intercalate. The thermal degradation of other intercalates Kln/a-SMA 95:5 and 90:10 are very similar to the thermal degradation of the Kln/a-SMA = 80:20 and not explicitly shown for clarity. Interestingly, the final degradation at 800 °C for the different intercalates Kln/a-SMA do no directly correspond to the original mixing ratios (e.g., 11% weight loss for Kln/a-SMA = 80:20, 18% weight loss for Kln/a-SMA = 70:30 ), as an indication that an improvement in thermal stability is obtained through interactions between both organic and inorganic phase. In parallel with observations be optical microscopy, the important differences in thermal stability of for ratios Kln/a-SMA = 50:50 to 5:95 can be explained by rather the formation of a continuous polymer phase surrounding the kaolinite platelets without intercalation, as demonstrated before. 

### 2.3. Imidization of Intercalated Kaolinite 

**Figure 8:**
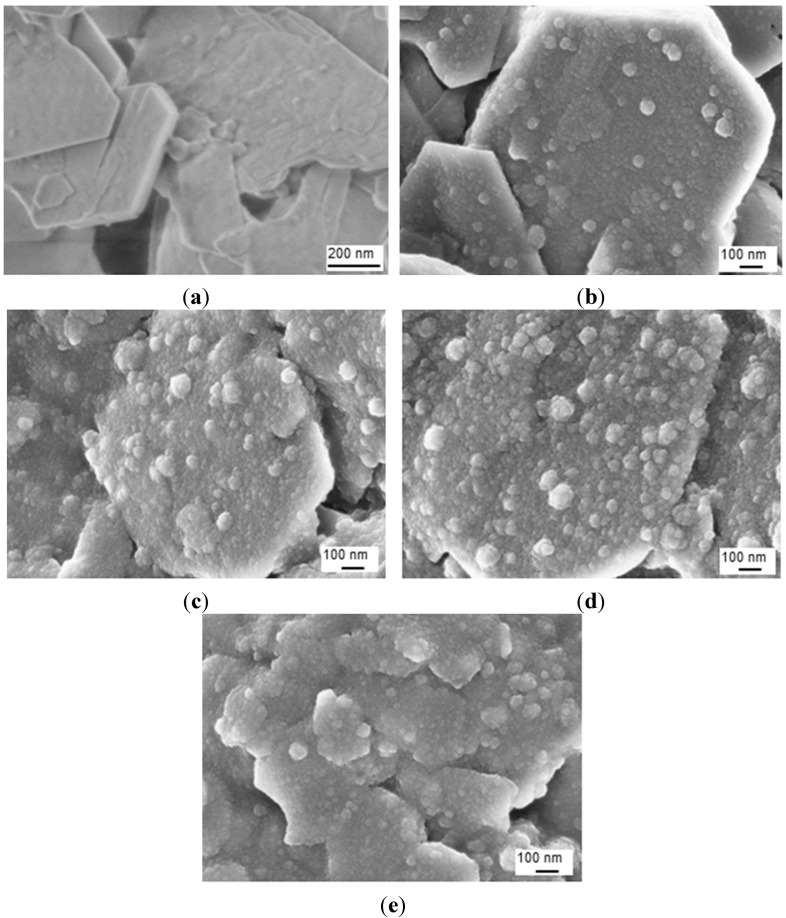
SEM images of surface-modified kaolinite nanocomposite platelets after chemical reaction for (**a**) pure Kln, (**b**) Kln/SMI = 95:5, (**c**) Kln/SMI = 80:20, (**d**) Kln/SMI = 70:30, (**e**) Kln/SMI = 50:50.

After imidization of the Kln/a-SMA in ratios 95:5 to 50:50, the homogeneous aqueous dispersions of surface-modified hybrid Kln/SMI platelets were obtained with white color and no tendency for separation or sedimentation of the modified kaolinite platelets over more than one week. The SEM images of the hybrid Kln/SMI platelets with different ratios of Kln/SMI = 95:5 to 50:50 are shown (Figure 8): the spherical SMI nanoparticles (20 to 60 nm average diameter) are preferentially deposited onto the hexagonal kaolinite platelets and the surface coverage of the platelets obviously increases at higher concentrations of a-SMA. The imidization of pure SMI nanoparticles typically results in the formation of spherical nanoparticles with 100 nm diameter, while the imidization in presence of inorganic particles results in smaller particle sizes. Although the large inorganic kaolinite platelets (>0.5 μm) generally have a tendency for settling in dispersion due to gravity, the precipitation of imidized nanoparticles on their surface contributes to stabilizing the dispersion. As such, the previously intercalated kaolinite may be further stabilized by the deposition of nanoparticles on their surfaces, increasing the surface hydrophobicity and lowering interactions between the exfoliated kaolinite layers. At other ratios of Kln/SMI below 50:50, however, a more continuous phase of separate pure SMI nanoparticles is formed as characterized previously [43], rather than SMI nanoparticle deposits on single kaolinite platelets. The latter can be explained by inappropriate intercalation of the Kln/a-SMA 50:50 compositions, as outlined before, and are not further considered.

The physical properties of aqueous dispersions of pure SMI nanoparticles and chemically reacted Kln/SMI nanocomposite particles are summarized (Table 2). Typically, the pH of the Kln/SMI dispersions is somewhat lower than for pure SMI nanoparticles due to a more important remaining fraction of ammonolyzed (*i.e.*, non-imidized) maleic anhydride in presence of kaolinite, as demonstrated below. Furthermore, the nanocomposite particle dispersions can be synthesized at a higher solid content and obtained slightly higher viscosity compared to the pure SMI nanoparticles, in parallel with the higher solid content that was obtained after previous synthesis of oil-filled SMI nanoparticles [52]. The slightly higher Zetapotential for nanocomposite particles also agrees with the previous synthesis of oil-filled SMI nanoparticles [52], and obtains a highest value at ratio Kln/SMI = 70:30. The stability of the aqueous dispersions are confirmed by a strongly negative Zetapotential that are attributed to charge effects near the carboxyl acid moieties that remain as a result of ring-opened cyclic anhydrides. In case of Kln/SMI nanocomposite particles, part of these charges may be additionally compensated through charge interactions between the ammonolyzed a-SMA and the kaolinite, as it was also observed in the presence of oil [52]: as a result, both the pH and Zetapotential (measured at the intrinsic pH of the dispersion) are affected. For the ratio Kln/SMI = 70:30, these interactions may be estimated to be at their maximum in respect with the lowest Zetapotential value. In the next paragraphs, the properties of the chemically imidized Kln/SMI nanocomposite platelets and physical mixtures of an aqueous kaolinite slurry with separately imidized pure SMI nanoparticles are compared. 

**Table 2:** Physical properties for aqueous dispersions of chemically reacted Kln/SMI.

Composition	pH	Solid content (wt %)	Viscosity (cp)	Zetapotential (mV)
Pure SMI	6.82	34.9	176	−60
Kln/SMI = 95:5	5.47	64.3	298	−58
Kln/SMI = 90:10	5.48	65.2	275	−58
Kln/SMI = 80:20	5.40	64.8	280	−55
Kln/SMI = 70:30	5.22	64.7	236	−48
Kln/SMI = 50:50	5.48	65.3	274	−55

**Figure 9:**
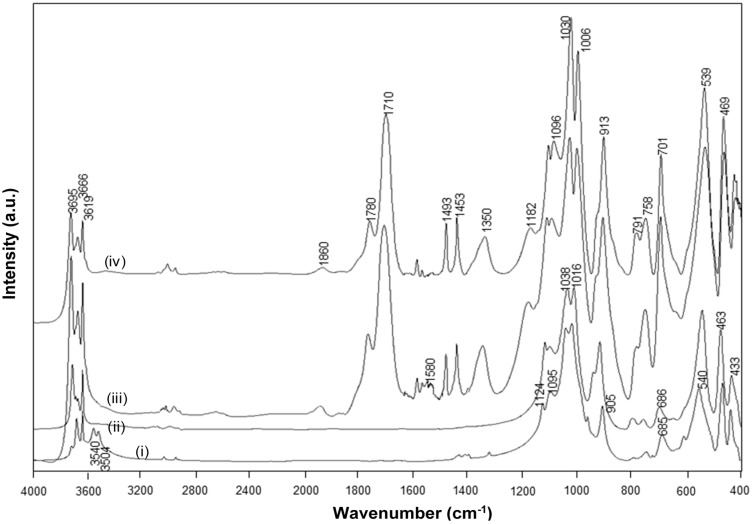
FTIR spectra for (**i**) Kln/a-SMA = 70:30 , (**ii**) Kln/a-SMA = 70:30 after thermal heating 120 °C for 1 h, (**iii**) Kln/SMI = 70:30 chemically reacted, (**iv**) Kln/SMI = 70:30 physically mixed.

The FTIR spectra for heat-treated Kln/a-SMA (1 h at 120 °C) before the imidization and Kln/SMI after the imidization (4 h at 160 °C), together with a spectrum of native Kln and pure SMI nanoparticles that were physically mixed in the same ratio Kln/SMI = 70:30, are compared (Figure 9). The spectral bands at 1493 and 1453 cm^−1^ corresponding to aromatic C–C stretch in styrene were used as reference bands. The absorption bands related to the inner-surface hydroxyl groups in kaolinite (3540 and 3504 cm^−1^) disappear after heat treatment, as an indication for hydrogen bonding between the functional groups in a-SMA and kaolinite after heating under mild temperatures up to 120 °C. The imidization reaction starts after heating under high temperatures of 160 °C, resulting in the appearance of two carbonyl stretching bands at 1780 cm^−1^ (in-phase carbonyl C=O stretching) and 1710 cm^−1^ (out-of-phase C=O stretching), characteristic for the imide I spectral fingerprint region [53]: the higher intensity of the imide I band at 1710 cm^−1^ relatively to 1780 cm^−1^ is typical for cyclic imides such as maleimide. Also, the imide III band at 1182 cm^−1^ develops as a shoulder band after imidization. In parallel, a small residual fraction of C=O in ring-opened anhydride remains existing (1860 cm^−1^). The differences between SMI nanoparticles that are physically mixed within the Kln/SMI or chemically imidized into Kln/SMI are observed in the wavenumber region 1600–1560 cm^−1^, which represents the formation of amic acid moieties (COOH / CONH_2_) as observed for maleic acid [54]. From FTIR spectra, the amic acid moieties form during imidization of a-SMA in presence of kaolinite, while they do not form during imidization of pure SMI nanoparticles that were later mixed with the kaolinite. In conclusion, the imidization reaction (ring-closure of ammonolyzed SMA) seems to be partly hindered in presence of kaolinite, which confirms the chemical interaction between the SMI nanoparticles and kaolinite after chemical imidization in addition to the previously demonstrated hydrogen bonding. 

**Figure 10:**
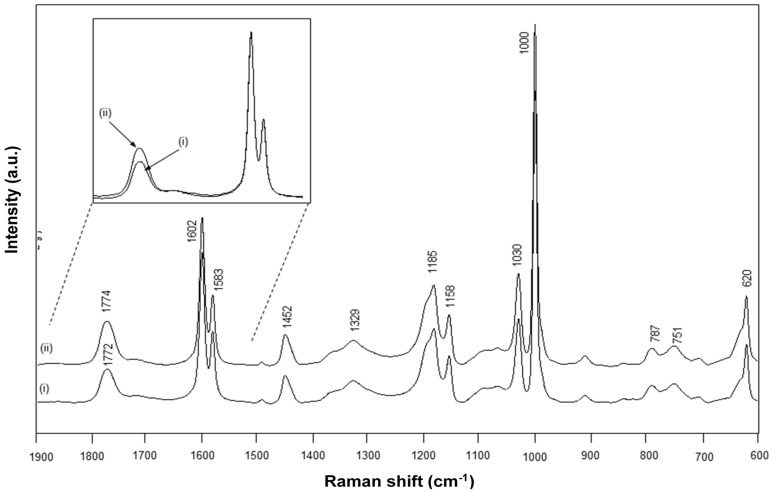
FT-Raman spectra for (**i**) chemically reacted Kln/SMI = 70:30, (**ii**) physically mixed Kln/SMI = 70:30.

The FT-Raman spectra for chemically imidized Kln/SMI and physically mixed Kln/SMI are compared (Figure 10), and also characterized by the existence of an imide absorption band at 1773 cm^−1^ (C=O, imide I) and 1329 cm^−1^ (C–N–C, imide II) [55]. Other bands correspond to the styrene aromatic C=C moieties as 1602, 1583, 1452 cm^−1^ and are used as reference bands for normalization. In the present concentration ratio Kln/SMI = 70:30, the intensity of kaolinite-related absorption bands at 800–700 cm^−1^ remain relatively low. This might indicate that the surface of the kaolinite platelets is effectively covered with SMI nanoparticles, forming the main Raman signal. For the same ratios for Kln/SMI, the imide I band for the chemically reacted Kln/SMI has a lower intensity than for physically mixed Kln/SMI. The lower imide intensity is in agreement with the higher amount of residual amic acid moieties previously observed by FTIR and its effects on the physical properties of Kln/SMI dispersions in previous Table 2. A band shift from 1772 cm^−1^ (chemical) to 1774 cm^−1^ (physical) can be explained as a change in resonance by stronger intermolecular interactions through hydrogen bonding with the imide groups.

### 2.4. Concentration Effects and Quantification

**Figure 11:**
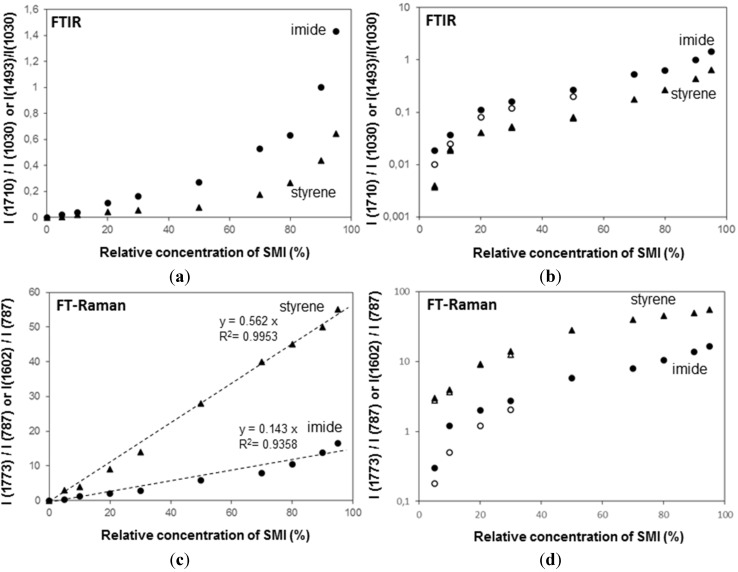
Concentration effects and quantification of band intensity ratios imide/kaolinite (●, ○), and styrene/kaolinite (▲, ○) from (**a**,**b**) from FTIR spectra, (**c**,**d**) FT-Raman spectra for physically mixed Kln/SMI (closed symbols) and chemically reacted Kln/SMI (open symbols), the chemically reacted Kln/SMI only for concentration ratios up to 50:50.

The FTIR spectra have been used for quantifying the relative concentration of SMI in physically mixed and chemically imidized Kln/SMI (Figure 11a,b), by plotting the height intensity of the imide-related absorption peak (1710 cm^−1^) or styrene-related absorption peak (1493 cm^−1^) relatively to the height intensity of the kaolinite-related absorption peak (1030 cm^−1^). For physically mixed Kln/SMI, the imide content does not evolve linearly with the concentration of SMI nanoparticles. On the other hand, it is interesting to note that the trend is almost linear for ratios of Kln/SMI = 100:0 to 50:50: as before, this is the most interesting concentration range for imidization with and optimum of Kln/SMI at around 70:30. The relative imide and styrene intensities for chemically imidized samples of Kln/SMI = 95:5 to 50:50 are compared in a logarithmic graph (Figure 11b), confirming that all chemically imidized Kln/SMI samples have lower imide content that the physically mixed ones, because of the previously explained interactions during imidization. The intensities for styrene are obviously the same for physical mixtures and chemical imidization, as the latter do not take part in the ammonolysis and imidization process. At the same time, the corresponding values of styrene for physical mixtures and chemically imidized Kln/SMI confirm that the ratios of kaolinite and organic copolymer were the same in both cases. 

The FT-Raman spectra have also been used for quantifying the amounts of imide, styrene and kaolinite (Figure 11c,d) by plotting the height intensity of the imide-related absorption peak (1773 cm^−1^) or styrene-related absorption peak (1602 cm^−1^) relatively to the height intensity of the kaolinite-related absorption peak (787 cm^−1^). The Raman intensity ratios provide a good linear relationship in the intensity ratios of imide (R^2^ = 0.94) and styrene (R^2^ = 0.99) and the relative intensities of imide are significantly less for chemically reacted SMI compared with physically mixed SMI. The imide content of the SMI nanoparticles can be calculated from Raman spectra, as the ratio of integrated peak areas of the imide I band (1773 cm^−1^) and styrene band (1602 cm^−1^), as calibrated before for the characterization of pure SMI nanoparticles [43]. Theoretically, the maximum imide content for a SMA copolymer with 26 mol % maleic anhydride is 26/(100 − 26) = 35%. The imide content of chemically reacted and physically mixed Kln/SMI is calculated (Table 3, see column non-annealed). For physically mixed Kln/SMI, it is evident that the imide content remains constant at around 27% and is similar to the properties of the pure SMI nanoparticles, as nanoparticles from a same batch have been used. The data also illustrate good reproducibility of the calculation method based on analysis of Raman spectra with a maximum variation of 0.5%. This implies that about 8% of the maleic anhydride moieties are not imidized and remain in the ammonolyzed state. The presence of non-imidized structures agrees with FTIR and provides good dispersion stability with Zetapotential values of about −60 mV. Otherwise, the imide content for chemically reacted Kln/SMI is about 20%–23% and is maximum for Kln/SMI = 70:30. The lower imide content for SMI synthesized in presence of inorganic materials illustrates that the kaolinite interferes with the imidization reaction and additional interactions between the a-SMA and the kaolinite (illustrated before as hydrogen bonding from FTIR analysis) prevents full imidization.

**Table 3:** Imide content (%) for chemically reacted and physically mixed Kln/SMI calculated from FT-Raman spectroscopy (relatively to a maximum of 35% for fully imidized SMI).

Composition	Physically mixed samples				Chemically reacted samples			
	Non-annealed	Thermally annealed			Non-annealed	Thermally annealed		
		135 °C	150 °C	180 °C		135 °C	150 °C	180 °C
Pure SMI	Samples were not mixed				27.0	33.8	30.2	29.2
Kln/SMI = 95:05	27.2	29.3	28.2	298.4	20.5	24.3	23.2	21.4
Kln/SMI = 90:10	26.8	30.3	29.1	27.8	20.3	24.8	22.8	21.3
Kln/SMI = 80:20	27.1	31.5	29.8	27.9	21.7	26.5	25.8	24.8
Kln/SMI = 70:30	27.0	33.2	31.8	29.8	23.0	29.5	27.4	26.4
Kln/SMI = 50:50	26.7	32.8	29.8	28.6	22.2	25.8	24.3	23.8
Kln/SMI = 30:70	27.2	31.2	29.5	28.5	Samples were not imidized			
Kln/SMI = 20:80	27.0	32.6	29.6	29.2				
Kln/SMI = 10:90	27.1	32.4	29.5	28.6				
Kln/SMI = 05:95	26.9	32.8	29.4	28.4				

### 2.5. Thermal Properties and Thermal Annealing Effect 

**Figure 12:**
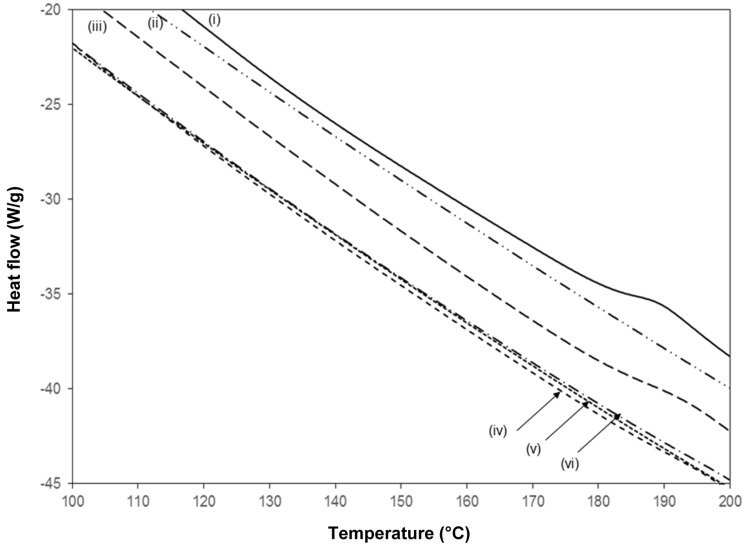
Detail of DSC curves during second heating run from 0–250 °C for (**i**) SMI, (**ii**) Kln, (**iii**) chemically reacted Kln/SMI = 70:30, (**iv**) physically mixed Kln/SMI = 70:30, (**v**) physically mixed Kln/SMI = 50:50, (**vi**) physically mixed Kln/SMI = 30:70.

A detail of the DSC heat flow curves for pure SMI together with chemically reacted and physically mixed Kln/SMI (Figure 12), indicates the position of the glass transition temperature *T*_g_ in the second heating cycle. No further transitions have been detected at temperatures below 100 °C and above 200 °C. The pure SMI nanoparticles have a clear glass transition at *T*_g_ = 185 °C, with a heat capacity change Δ*c*_p_ = 0.3392 J/(g°C). It is known that pure kaolinite does not show a transition temperature in the considered temperature interval, while the lack of any transition indicates that DMSO has been fully eliminated [27]. The chemically reacted Kln/SMI with ratio 70:30 shows a single glass transition temperature at *T*_g_ = 192 °C, with a heat capacity change Δ*c*_p_ = 0.092 J/(g°C). Compared with pure SMI, the slightly higher *T*_g_ for Kln/SMI might indicate that the molecular mobility of SMI nanoparticles is somewhat hindered due to good attachment of the nanoparticles to the kaolinite surface by hydrogen bonding. The smaller heat capacity change Δ*c*_p_ quantitatively corresponds to the presence of about 30% SMI nanoparticles and 70% kaolinite: the heat capacity change Δ*c*_p_ = 0.092 J/(g°C) for chemically reacted Kln/SMI is about 27% of the value for pure SMI, as the imide content for Kln/SMI is lower than for pure SMI. The absence of any further transition temperature in chemically reacted Kln/SMI illustrates that no residual DMSO has been detected, which would have transition temperatures at 117 and 173 °C. In case of physically mixed Kln/SMI, no clear transition temperatures are observed for any of the mixing ratios, as the thermal transition temperatures remain determined by the kaolinite.

**Figure 13:**
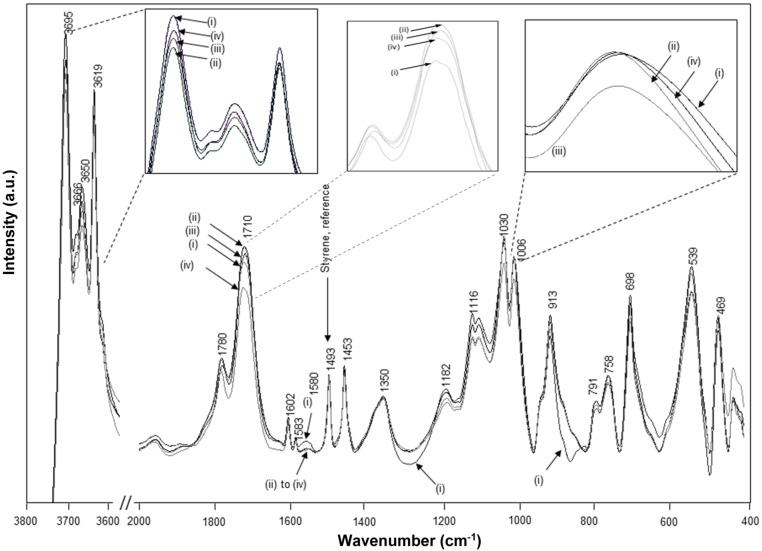
FTIR spectra of chemically reacted Kln/SMI = 70:30 after thermal annealing at different temperatures, (**i**) non-annealed, (**ii**) 135 °C, (**iii**) 150 °C, (**iv**) 180 °C.

The chemically reacted Kln/SMI has been thermally annealed at 125, 135 and 180 °C for 6 h, resulting in further modifications in the FTIR spectra (Figure 13). The variations in intercalated kaolinite structure upon curing are inferior, in parallel with the lack of transitions noticed in TGA and DSC. Most variations are observed at around the characteristic SMI absorption bands. The remaining parts of ammonolyzed SMA gradually disappear under thermal curing by further condensation into imide, which is characterized by disappearance of absorption band related to amic acid (1580 cm^−1^). The additional formation of imide structures upon thermal curing is most favoured at 135 °C, with a maximum intensity of the imide I band (1710 cm^−1^) in parallel with a red-shift associated with C=O structures from 1712 cm^−1^ (before curing) to 1705 cm^−1^ (135 °C) and 1708 cm^−1^ (180 °C). The observed band shift correlates to hydrogen bonding between the SMI and kaolinite layers. Moreover, two characteristics bands for the aromatic structure in styrene are sensitive to thermal curing: the shoulder band at 875 cm^−1^ was not present in the initial Kln/SMI but increases in intensity after thermal curing, and the band at 1000–1006 cm^−1^ has blue-shifted in position with a maximum frequency after curing at 135 °C. The latter can be related to reorientations of the styrene rings and the blue-shifts of hydrogen bonds are indicative for interactions that lead to compression of the bond length [56]. The FTIR bands for styrene may partly overlap with kaolinite-related absorption bands at 1010 and 1030 cm^−1^, corresponding to the in-plane Si–O–Si stretching vibrations that are also blue-shifted by intercalation [44]. The latter band shifts were not observed as pronounced during the thermal curing of pure SMI nanoparticles [57], and clearly indicate constraint interactions in presence of kaolinite. The hydroxyl bonding of the nanoparticles to the kaolinite improves after curing at 135 °C, with a minimum intensity of the 3695 cm^−1^ band.

**Figure 14:**
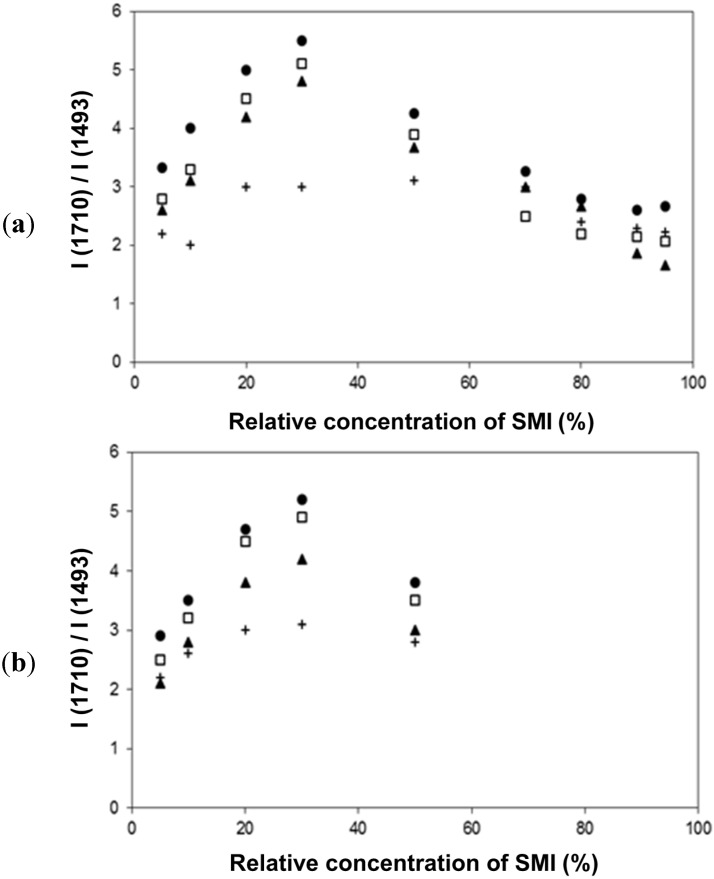
Thermal curing effects and quantification of band intensity ratios (imide/styrene) from FTIR spectra, (**a**) physically mixed Kln/SMI, (**b**) chemically reacted Kln/SMI: (+) non-annealed room temperature, (●) 135 °C, (□) 150 °C, (▲) 180 °C.

The relative intensities of imide-related absorption bands from FTIR spectra as a function of the curing temperature for physically mixed Kln/SMI and chemically reacted Kln/SMI are further compared (Figure 14), in order to follow the variations in imide structure during thermal curing. For both physically mixed and chemically modified Kln/SMI, a maximum intensity of imide structures develops after curing for 6 h at 135 °C for a ratio Kln/SMI = 70:30. Once again, these findings illustrate that the formulation of Kln/SMI nanocomposite platelets is optimized at a concentration ratio of Kln/SMI = 70:30. The possibility for obtaining a maximum imide content after curing illustrates that the interactions between both components is optimized at this concentration ratio. The evolution in imide content after curing is confirmed by quantitative calculations from Raman spectra (Table 3, part thermally annealed), indicating a maximum imide content of 29.5% for chemically reacted Kln/SMI = 70:30 after curing at 135 °C. The calculations of imide content after curing the physically mixed Kln/SMI is higher, and rather reflect the same evolution that was previously obtained after curing the pure SMI nanoparticles [57]. Theoretically, the imide content increases after thermal curing due to further polycondensation of the ammonolyzed (ring-opened) maleic anhydride into the ring-closed imide structure. For the physically mixed Kln/SMI, this imidization reaction seems not hindered by additional interactions between the SMI and the kaolinite. For the chemically reacted Kln/SMI, this imidization reaction is hindered by additional interactions between the kaolinite and the a-SMA, which hinders the further imidization.

For future practical applications as hydrophobic coating additives, it has been illustrated for pure SMI nanoparticles that a maximum imide content correlates with maximum hydrophobicity [57]. The possibility to tune the imide content of the modified kaolinite platelets makes them ideal candidates for filler agents that can be added into hydrophobic coatings, where the hydrophobicity can theoretically be tuned in parallel with the imide content by thermal curing. As specific additional benefits in adding Kln/SMI fillers compared with pure SMI nanoparticles into a coating formulation, the retention of the SMI nanoparticles at the coating surface is enhanced and the creation of a hierarchical roughness with a microscale component of the exfoliated Kln platelets and a nanoscale component of the SMI nanoparticle deposits may further augment the hydrophobicity of hydrophobic surfaces. The full study on further applications of the kaolinite platelets in paper coatings, however, is outside the direct scope of this paper.

## 3. Experimental Section 

### 3.1. Materials 

Pellets of poly(styrene-*co*-maleic anhydride) or SMA with molecular weight *M_w_* = 80.000 g/mol and 26 mol % maleic anhydride (MA) were obtained from Polyscope (Geleen, The Netherlands). Dry kaolinite (Kln) powder with particle diameters <2 μm and aspect ratio 20:1 was obtained from Imerys (Paris, France) and used without further treatment. Ammonium hydroxide was obtained from Belgocare (Niel, Belgium) as a 25% aqueous solution (0.9 g/mL). Other solvents such as dimethylsulfoxide (DMSO) with analytical reagent grade were obtained from Sigma Aldrich.

### 3.2. Procedure for Intercalation

In a first step, the kaolinite was subjected to intercalation with ammonolysed SMA (a-SMA) by guest displacement of DMSO molecules. The a-SMA was prepared by mixing SMA pellets with ammonium hydroxide in a ratio of NH_3_/MA = 1.01 relatively to the maleic anhydride moieties and stirring the solution for 1 h at 90 °C. The pre-intercalated kaolinite was prepared by adding 0.5 g kaolinite in a solution of either pure DMSO (20 ml) or a mixture of DMSO + water (19.6 mL/0.4 mL) and sonication treatment for 10–20 h at 40 °C. The pre-intercalates were washed several times with pure water to remove the excess DMSO and dried in vacuum at 80 °C overnight. The pre-intercalates of Kln/DMSO + water were subsequently mixed with a-SMA in different amounts and the mixtures were sonicated for another 10 h at 40 °C. The concentration ratios (w/w) of Kln/a-SMA were varied from 95:5, 90:10, 80:20, 70:30, 50:50, 30:70, 20:80, 10:90 to 5:95. The solutions of Kln/a-SMA were finally dropped into methanol and precipitated to yield a powder, which was washed several times with fresh water until no residual solvents or excess a-SMA were detected and a clear white powder was obtained. The resulting powders of Kln/a-SMA were dried at 80 °C in a vacuum oven for 24 h. As a reference sample Kln/a-SMA*, the a-SMA was directly added to a dispersion of kaolinite in water at a ratio Kln/a-SMA = 70:30 and sonicated for 10 h at 40 °C, without pre-intercalation of the kaolinite with DMSO + water.

### 3.3. Procedure for Imidization

In a second step, the Kln/a-SMA was further reacted during an *in-situ* imidization reaction of the ammonolyzed SMA into poly(styrene-*co*-maleimide) or SMI nanoparticles. The aqueous dispersions of hybrid Kln/SMI nanocomposite particles were prepared at a solid content of 65 wt % with concentrations ratios (w/w) of Kln/a-SMA = 95:5, 90:10, 80:20, 70:30 and 50:50. The lower ratios of Kln/a-SMA were not imidized as the single kaolinite surfaces were not efficiently modified. The imidization reaction was done by heating the aqueous reaction mixture in a laboratory autoclave (1 L) for 4 h to 160 °C at a pressure of 6 bar, under continuous stirring at 400 rpm. The reaction mixture was finally cooled down to room temperature and upon evacuation from the autoclave reactor, a homogeneous aqueous dispersion of Kln/SMI without any phase separation between kaolinite platelets and SMI nanoparticles was obtained. The dispersions remained stable for over one year. 

In a reference experiment, pure a-SMA was imidized under the same conditions (NH_3_/SMA = 1.01, 160 °C, 6 bar), resulting in a homogeneous aqueous dispersion of pure SMI nanoparticles, in parallel with previous experiences [43]. The latter SMI dispersions were mixed with pure kaolinite in ratios of Kln/SMI = 95:5 to 5:95. The so-called “physically mixed Kln/SMI” was prepared in different ratios by magnetic stirring for 1 h at 30 °C followed by sonication for 3 h at 30 °C. The pure SMI and physically mixed Kln/SMI were further analyzed and compared against the chemically reacted Kln/SMI.

### 3.4. Characterization Methods

The dispersions of Kln/SMI were characterized by measuring pH (Knick 752 Cl), viscosity (Brookfield, DV-II Pro) and Zetapotential (Zetasizer Nano ZS, Malvern). The XRD analysis of dried powders was performed on a Philips PW3710 diffractometer with CuK α radiation (mean wavelength 0.15418 nm), using a generator voltage of 45 kV and generator current of 40 mA. Fourier transform infrared spectroscopy (FTIR) was done on a Spectrum GX System (Perkin Elmer), embedding the dried powders in KBr pellets. The spectra were collected in between 4000–400 cm^−1^ at a 2 cm^−1^ resolution and averaged over 32 scans. Fourier-transform Raman (FT-Raman) spectroscopy was done on the same instrument. The spectra were averaged over 64 scans with a Nd:YAG laser power of 500 mW at a 2 cm^−1^ resolution between 4000 and 100 cm^−1^. The thermal stability was determined by thermogravimetric analysis (TGA, Mettler Toledo SDTA851) on a sample of about 12 mg, applying a heating rate of 20 °C/min in air between 23 and 1000 °C. Differential scanning calorimetry (DSC) was done on a sample size of 5 mg heated under nitrogen atmosphere at 10 °C/min over two cycles from 23–250 °C, using a Q2000 equipment (TA Instruments, Zellik, Belgium). Optical microscopy (Olympus BX51) was made by drying the dispersions on a glass plate. For transmission electron microscopy (TEM, Leo 912 Omega, Zeiss), the dispersions were diluted 100 times and deposited on a carbon-grid and air-dried for evaluation. The scanning electron microscopy (SEM XL30, Philips) of the Kln/SMI particles was made by drying the dispersions on a glass plate. 

## 4. Conclusions 

In a first step, the intercalation of kaolinite (Kln) by ammonolysed high-molecular weight poly(styrene-co-maleic anhydride) or a-SMA by guest displacement of dimethylsulfoxide (DMSO) was demonstrated. The pre-intercalant of Kln/DMSO + water could be formed within reasonable reaction times by adding some water. Based on FTIR evaluations, only partial intercalation of the pre-intercalant has been obtained for the studied reaction times, while this seemed effective under reasonable reaction times for further intercalation of the a-SMA. In contrast, the direct intercalation of a-SMA in pure kaolinite was not possible and the pretreatment with DMSO + water for 10 h was necessary. At an optimized ratio of Kln/a-SMA = 70:30, the intercalation ratio of 93% and interlayer distance of 1.234 nm are maximized according to XRD data. The expansion of the layered kaolinite structure by a-SMA has been effectively demonstrated by variations in the kaolinite lattice structure and O–H stretching according to FTIR and FT-Raman spectroscopy. The displacement of DMSO by a-SMA results in better thermal stability of the intercalated Kln/a-SMA compared with the Kln/DMSO + water pre-intercalate. According to these findings, an appropriate pre-intercalation of the kaolinite is necessary and has been optimized for good intercalation and exfoliation in presence of a-SMA, in contrast with direct the imidization of a-SMA with a kaolinite slurry.

In a second step, the chemical imidization of intercalated Kln/a-SMA results in the further exfoliation of kaolinite in parallel with the surface deposition of poly(styrene-co-maleimide) or SMI nanoparticles on the surfaces of the kaolinite layers. As confirmed by XRD and microscopy, the exfoliation and deposition is optimized at ratios of Kln/SMI = 70:30. The imide content of Kln/SMI is evidently lower than pure SMI nanoparticles, as the presence of inorganic carrier materials interferes with the imidization process and results in detectable amounts of amic acid by FTIR. The latter disappears by additional thermal curing at 135 °C, maximizing the imide content of the Kln/SMI nanocomposite platelets at concentration ratios of Kln/SMI = 70:30. The properties of physical mixtures of Kln/SMI are different from the chemically modified Kln/SMI, as no exfoliation and higher imide contents are observed for physical mixtures. Therefore, the chemical imidization in presence of a-SMA and kaolinite clearly favours chemical interactions between both components. The quantification of Kln/SMI nanocomposites can be based on FT-Raman spectra, indicating linear concentration dependency and quantitative data for imide content calculations. Finally, the presented route for formulation of kaolinite nanocomposite platelets by intercalation, exfoliation and surface modification with organic nanoparticles results in stable aqueous dispersions with 65 wt % solid content. 

## Supplementary Materials

Supplementary materials can be accessed at: http://www.mdpi.com/1996-1944/8/7/4363/s1.
