# Supplementary material for: Kaolinite Nanocomposite Platelets Synthesized by Intercalation and Imidization of Poly(styrene-co-maleic anhydride)
Source: Materials (Basel). 2015 Jul 16;8(7):4363–88. doi: 10.3390/ma8074363 (PMC5455644; doi:10.3390/ma8074363)
Supplement: Supplementary file 1 [file materials-08-04363-s001.pdf]

Supplementary Information

Detailed description of the FTIR spectral data (Figure 5).

The O–H stretching region for kaolinite (Figure 5a) has typically four FTIR bands at 3695, 3667, 3652 and 3620 cm^{-1} [1]. The band at 3620 cm^{-1} represents the stretching frequency of internal hydroxyl groups and is not influenced by interlayer modifications: the inner hydroxyl groups are resistant to exchange with intercalants and dehydroxylation because of its recessed location within the kaolinite structure. Therefore, the 3620 cm^{-1} band can be selected as a reference band in this spectral range. The three remaining bands at 3695, 3667 and 3652 cm^{-1} are related to the hydroxyl groups present on the interlayer kaolinite surfaces and are referred to as inner-surface hydroxyl groups: they are accessible for hydrogen bonding with intercalated guest molecules [2,3], and consequently shift upon intercalation [4]. The 3695 and 3668 cm^{-1} bands have been assigned to the stretching vibration of two coupled inner-surface hydroxyl groups oriented perpendicular to the (001) plane; the 3652 cm^{-1} band has been assigned to the stretching motion of an inner-surface hydroxyl group almost parallel to the interlayer surface [5]. The spectral bands for Kln/DMSO after 10 h are similar to Kln and indicate almost no intercalation, while very slight variations show up after 20 h of treatment. The spectral bands for Kln/DMSO + water after 10 h show weak variations as seen in other studies [6]: there is a slight shift from 3695 to 3697 cm^{-1} and 3620–3622 cm^{-1} while the intensity for the 3697 cm^{-1} band has been diminished, as an indication of slight changes in the interlayer conformation and hydrogen bonding between the hydroxyls on the kaolinite surface. This illustrates that the partial intercalation with DMSO + water over short times remains limited in agreement with earlier studies running over 60 days [6], but the aid of water is effective to accelerate the intercalation. For the Kln/a-SMA* reference sample, the characteristic absorption bands of the native kaolinite structure have not changed. Otherwise, the pattern of the O–H stretching region for Kln/a-SMA intercalates differs much more strongly from both Kln and Kln/DMSO and corresponds to the structure of intercalated kaolinite with main absorption bands at 3695, 3660, 3620, 3540 and 3504 cm^{-1}. Although the Kln/DMSO + water pre-intercalates did not fully develop after a limited treatment time of 10 hours (in agreement with XRD characterization), the presence of DMSO as a pre-intercalate was necessary for successful intercalation with a-SMA. The strong decrease in intensity of the 3695 cm^{-1} band illustrates that the hydroxyl groups at the outer surface are occupied through interactions with a-SMA: this band disappears almost completely for a ratio Kln/a-SMA = 70:30 as it represents an optimum intercalation concentration with high interactions between the kaolinite and a-SMA. The development of a new band at 3660 cm^{-1} can be attributed to the formation of a complex between the inner surface hydroxyl groups and the a-SMA. In parallel, the two bands at 3540 and 3504 cm^{-1} can be attributed to the formation of hydrogen bonding between the inner-surface hydroxyl groups of the kaolinite layers and the intercalated a-SMA. After intercalation with a-SMA, the wavenumber differences between 3695–3660 cm^{-1} and 3540–3504 cm^{-1} are almost similar (35–36 cm^{-1}) and both bands may correspond to the stretching frequencies of coupled inner-surface hydroxyl groups perpendicular to the (001) plane that are hydrogen bonded to intercalated a-SMA.

The lattice vibrations for kaolinite (Figure 5b) are characterized by two main FTIR bands at 1010 and 1030 cm^{-1}, corresponding to the in-plane Si–O–Si stretching vibrations that are often blue-shifted by intercalation [7]. The two bands become more distinguishable and are most intense for the pre-intercalant Kln/DMSO + water and for intercalated Kln/a-SMA = 70:30. In parallel, they have significantly shifted to 1012 and 1032 cm^{-1} respectively, for Kln/DMSO + water, and shifted further to 1015 and 1035 cm^{-1},

respectively, for Kln/a-SMA. The latter indicates a progressive intercalation of the high-molecular a-SMA as it was also observed for intercalation of other macromolecules [8]. These absorption bands start to flatten at ratios of Kln/a-SMA = 50:50 and indicate that a further increase in a-SMA concentrations to 50:50 and 5:95 was not successful in agreement with the previous XRD data (Table 2): the ratios Kln/a-SMA = 50:50 to 5:95 are therefore not further detailed. The band at 1110 cm^{-1} corresponding to perpendicular Si–O vibrations, remains existing after pre-intercalation of Kln/DMSO + water, while the band becomes more perturbed by further intercalation of a-SMA in different ratios: then, it shifts to 1116 cm^{-1} together with the appearance of a new band at 1098 cm^{-1}. The latter band at 1098 cm^{-1} starts to develop slightly for the Kln/DMSO + water pre-intercalate and is fully present for Kln/a-SMA with a maximum intensity at the ratio 70:30, as an indication for large disturbance of the silicate surface near the oxyethylene groups at this particular ratio Kln/a-SMA. The Al–O–H bending motion of inner-surface hydroxyl groups in kaolinite is observed at 914 and 938 cm^{-1} [9]. The band at 914 cm^{-1} characterizes the bending vibrations of Al–O–H inner hydroxyls groups that are inaccessible [10,11]: while remaining constant for Kln/DMSO and Kln/DMSO + water, it shifts towards 905 cm^{-1} due to repulsive interactions between the silica $(SiO)_6$ macro-ring of the silicate surface and the internal hydroxyl of kaolinite by the intercalant [12,13]. The band at 938 cm^{-1} characterizes the in-plane bending of the Al–O–H inner-surface hydroxyl groups [10,11]: it lowers in intensity upon intercalation with DMSO + water, while it has completely disappeared for intercalated Kln/a-SMA. It complements the previously described decrease in inner-surface hydroxyl stretching bands and was also confirmed for other intercalants [6]. The presence of small peaks at 1430, 1404 and 1316 cm^{-1} has previously also been observed for intercalated kaolinite [14]. The new bands at 957 and 607 cm^{-1} are due to the presence of a-SMA. All of the previous FTIR observations are in agreement with the complete displacement of the DMSO molecules in the interlayer space and replacement by a-SMA macromolecules that bind to the kaolinite surface by H-bonds with the inner-surface hydroxyls.

References

1. Van der Marel, H.W.; Krohmer, P. O–H stretching vibrations in kaolinite, and related minerals. *Contr. Mineral. Petrol.* **1969**, *22*, 73–82.
2. Forst, R.L.; Kristof, J.; Paroz, G.N.; Kloprogge, J.T. Molecular structure of dimethyl sulfoxide intercalated kaolinites. *J. Phys. Chem. B* **1998**, *102*, 8519–8532.
3. Frost, R.L.; Kristof, J.; Horvath, E.; Kloprogge, J.T. Molecular structure of dimethyl sulfoxide in DMSO-intercalated kaolinites at 298 and 77 K. *J. Phys. Chem. A* **1999**, *103*, 9654–9660.
4. Zhang, S.H.; Qiang, Y.H.; Ou, X.M.; Xia, H. FTIR and XRD analysis studies of intercalation of kaolinite with benzamide. *Spectrosc. Spectr. Anal.* **2009**, *29*, 2067–2070.
5. Rouxhet, P.G.; Samudacheata, N.; Jacobs, H.; Anton, O. Attribution of the OH stretching bands of kaolinite. *Clay Miner.* **1977**, *12*, 171–179.
6. Johnston, C.T.; Sposito, G.; Bocian, D.F.; Birge, R.R. Vibrational spectroscopic study of the interlamellar kaolinite-dimethyl sulfoxide complex. *J. Phys. Chem. A* **1984**, *88*, 5959–5964.
7. Frost, R.L. Hydroxyl deformation in kaolins. *Clays Clay Miner.* **1998**, *46*, 280–289.

8. Zhang, S.; Liu, Q.; Cheng, H.; Zeng, F. Combined experimental and theoretical investigation of interactions between kaolinite inner surface and intercalated dimethyl sulfoxide. *Appl. Surf. Sci.* **2015**, *331*, 234–240.
9. Farmer, V.C.; Russell, J.D. The infra-red spectra of layer silicates. *Spectrochim. Acta* **1964**, *20*, 1149–1173.
10. Frost, R.L.; Kristof, J.; Mako, E.; Kloprogge, J.T. Modification of the hydroxyl surface of potassium acetate intercalated halloysite between 25 and 300°C. *Am. Miner.* **2000**, *85*, 1735–1743.
11. Costanzo, P.M.; Giese, R.F. Dehydration of synthetic hydrated kaolinites: A model for the dehydration of halloysite (10A). *Clays Clay Miner.* **1985**, *33*, 415–423.
12. Duer, M.J.; Rocha, J.; Klinowski, J. Solid-state NMR studies of the molecular motion in the kaolinite: DMSO intercalate. *J. Am. Chem. Soc.* **1992**, *114*, 6867–6874.
13. Raupach, M.; Barron, P.F.; Thompson, J.G. Nuclear magnetic resonance, infrared, and X-ray powder diffraction study of dimethylsulfoxide and dimethylselenoxide intercalates with kaolinite. *Clays Clay Miner.* **1987**, *35*, 208–219.
14. Tunney, J.J.; Detellier, C. Preparation and characterization of two distinct ethylene glycol derivatives of kaolinite. *Clays and Clay Minerals* **1994**, *42*, 552–560.
